# PhenoTimer: Software for the Visual Mapping of Time-Resolved Phenotypic Landscapes

**DOI:** 10.1371/journal.pone.0072361

**Published:** 2013-08-12

**Authors:** Maria Secrier, Reinhard Schneider

**Affiliations:** 1 Structural and Computational Biology Unit, European Molecular Biology Laboratory (EMBL), Heidelberg, Germany; 2 Bioinformatics Core Facility, Luxembourg Centre for Systems Biomedicine (LCSB), University of Luxembourg, Esch sur Alzette, Luxembourg; University of Rome, Italy

## Abstract

Timing common and specific modulators of disease progression is crucial for treatment, but the understanding of the underlying complex system of interactions is limited. While attempts at elucidating this experimentally have produced enormous amounts of phenotypic data, tools that are able to visualize and analyze them are scarce and the insight obtained from the data is often unsatisfactory. Linking and visualizing processes from genes to phenotypes and back, in a temporal context, remains a challenge in systems biology. We introduce PhenoTimer, a 2D/3D visualization tool for the mapping of time-resolved phenotypic links in a genetic context. It uses a novel visualization approach for relations between morphological defects, pathways or diseases, to enable fast pattern discovery and hypothesis generation. We illustrate its capabilities of tracing dynamic motifs on cell cycle datasets that explore the phenotypic order of events upon perturbations of the system, transcriptional activity programs and their connection to disease. By using this tool we are able to fine-grain regulatory programs for individual time points of the cell cycle and better understand which patterns arise when these programs fail. We also illustrate a way to identify common mechanisms of misregulation in diseases and drug abuse.

## Introduction

From subcellular to population level, dynamic phenomena resulting from a combination of genetic and environmental factors shape diversity in wide arrays of phenotypes. The connection between the genotype and the phenotype is of great interest to researchers, as it can give clues into healthy and perturbed states, eventually leading to the differential treatment of diseases. Placing this in a temporal context enables us to better understand developmental features and triggers of disease onset and progression.

In recent years, the high-throughput sequencing technology has delivered increasing amounts of data on this type of studies [1–4]. Along with it, we are also witnessing a deluge of imaging data originating from large-scale phenotypic screens [5–8]. Despite the progress made in techniques to collect data at finer spatial and temporal resolution, a major bottleneck remains the interpretation of the results. In this respect, visualization constitutes a good aid in emphasizing important features of the data. However, in the growing landscape of large-scale multidimensional phenotypic datasets, visualization tools can hardly cope with the amount of data delivered by these methods, especially when the data is temporally stratified.

Time-focused visualization has been the object of different tools, like GATE [9], VistaClara [10], BioTapestry [11], TVNViewer [12], Arena3D [13,14], iPath [15] or iTOL [16]. These tools provide functionality for analyzing gene regulatory, metabolic, or protein–protein interaction networks that change with time. They use visual queues like color shifts, dynamic linking or embedding of heat maps or bar charts to depict these changes. However, there is limited attempt to compare phenotypic outcomes timewise, as reflected by genetic factors. Time thus remains a challenging aspect in this area. With the increasing amount of large-scale time-resolved genotype-phenotype data, a new approach to time-resolved phenotypic visual inspection is needed.

We introduce PhenoTimer, an open source tool for the visualization of time-driven phenotypic relationships in a genetic context. By using a novel combination of 2D/3D temporal projection displays and 2D network visualization, it enables the dynamic capturing of key points of biological processes. Temporal gene-phenotype connections can be analyzed in an interactive manner for link discovery and hypothesis generation. PhenoTimer is available for download as a standalone application from http://phenotimer.org/, along with the source code, the files used for the examples in this paper and other sample files for testing.

## Materials and Methods

### Availability

PhenoTimer was developed using Processing 1.5.1 (http://processing.org/), a Java-based environment with OpenGL integration. It runs on Mac OSX, Windows and some Linux environments (Ubuntu 9.04, limited testing). PhenoTimer is open source and freely available for academic use under the GNU GPL v 3.0 license at the following website: http://phenotimer.org/. The Java Run time Environment version 1.6 or higher (http://www.java.com/) is needed to run the tool. Mac users should also install the JOGL libraries (http://opengl.j3d.org/).

### Implementation

PhenoTimer uses 2D and 3D temporal projections to track connections between different phenotypes. These connections underline common genetic factors through time. The purpose is to explore the genetic-phenotypic space from a different perspective: how are two phenotypes similar, how do they relate to each other and what common genetic mechanisms govern the two biological processes? It also looks at how phenotypic traits can evolve successively from previous traits and how networks come into play in this progression.

**Figure 1:**
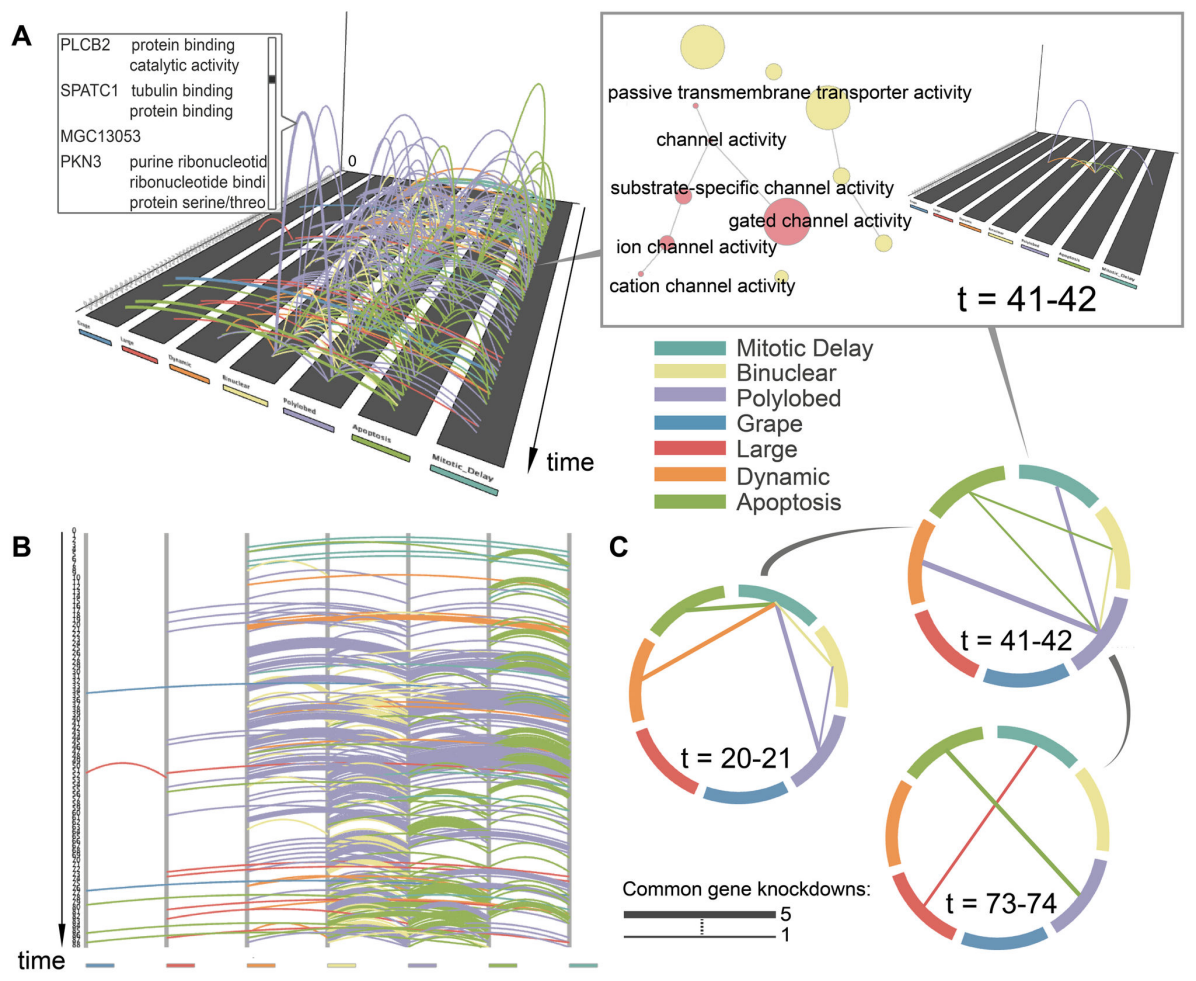
Phenotypic transition patterns in cell populations upon knockdown of genes essential for cell division. Three arc representations are shown: (A) 3D; (B) linear 2D; (C) circular 2D. An arc represents a transition from one phenotype to the other at consecutive time points. The color of the arc corresponds to the phenotype into which the cells transition. The height (3D) and width (2D), respectively, of arcs indicates the number of genes whose suppression causes the respective phenotypic transition at that particular moment (at most 5 genes for this dataset). The GO term network in the boxed picture in the upper right corner highlights (in red) the molecular functions of the genes whose knockdown causes a transition at time point 41. The respective transitions are shown as arcs in the plot for the particular time point. The size of the nodes in the network is proportional to the number of genes in the dataset that are enriched for the respective function. The GO network was generated using BiNGO [37] and subsequently loaded into PhenoTimer.

The main novelty of this tool consists in visualizing connections between phenotypes in the form of arcs linking the respective phenotypes for every time point or for a time interval. We use 3D, linear 2D and circular 2D projections to represent these arcs (see Figure 1 and Figure S1A–C). Heat maps and timeline plots of gene vector values for each phenotype complement these depictions, as detailed in Figure S1D and S1E. As such, several view modes are available to the user: (a) the 3D arc view (default, with connections through time represented as arcs in three dimensions); (b) the 2D arc view (a flattened view of the previous representation, for disambiguation); (c) the circular view (with phenotypes arranged as segments of a circle); (d) the heat map view; and (e) the line plot view (the last two both with global and zoomed-in views at each time point). One can use the view modes complementarily, depending on the size of the dataset and the topic addressed. For more details on the comparative strengths and weaknesses of each view, see Table S1.

#### The arc representation

The “connection”, represented as an arc, can be defined to suit the particular biological question under investigation. For instance, a “connection” between two phenotypes can indicate that these phenotypes are the result of disrupting some genes involved in the same pathway or process or it can highlight a transition between these two phenotypes for at least one genetic event (gene suppression, overexpression etc.). The color of the arcs codes for the directionality of the connection where needed (like in the case of transitions from one phenotype to the other), corresponding to the end phenotype. In case there is no directionality associated to the link, all connections can be set to a single color. The height (for 3D) or the width (for 2D) of the arc is proportional to the number of genes or gene-linked events for which that connection appears at that particular time point.

The reason for offering three types of depiction for the phenotypic connections is that, depending on the size and content of the dataset, one or the other visual representation may prove more useful in detecting patterns in the data. The height of arcs in the 3D view is better distinguishable than the width in the 2D layout in the case of overlapping arcs, thus acting as a more efficient indicator of the number of genes involved in the relationship. However, 3D layouts have been shown to be misleading, major issues referring to occlusion and perspective distortion [17]. While the zoom, pan and rotating capabilities partially overcome this, the 2D linear and circular representations prove more effective in surmounting these potential pitfalls. The 2D linear layout is adequate in looking at time traces of connections. The 2D circular layout, similar to Circos [18] or TVNViewer [12], serves well for individual time point analysis.

Some of the design choices are similar to the ones described by 19. In this paper, the authors use 3D links to highlight gene combinations that may be biologically relevant based on their expression, on a heat map of microarray values. In contrast, PhenoTimer looks at connections between phenotypes rather than single genes, and offers more flexibility in observing features by switching between 3D and 2D views, while at the same time allowing for further data integration.

To minimize clutter, we reorder the phenotypic lanes for optimal viewing of connections. To this purpose, we use an agglomerative hierarchical clustering algorithm [20] to maximize the number of links between two adjacent phenotypes.

Thresholds can be set for phenotypes and the time offset, in order to filter the dataset for relationships of interest or at different time intervals (Figure S2C and S2E, respectively). Importantly, if no thresholds are set for the phenotypes, all phenotypes will appear interconnected. One must filter for phenotype-associated values that reflect outstanding gene behavior (e.g. lowly/highly expressed). For the time offset, connections between phenotypes from time point *t* to time point *t*+*x* will be shown, where *x*∈{0,*n*} is the time offset, *n* being the total number of time points.

Bar charts can be loaded and visualized in the 3D arc mode in parallel to the phenotypic connections, as shown in Figure S1A. These usually contain measurements of some parameter associated to individual time points (e.g. the number of genes that are expressed at a particular moment). The user can decide on the data that makes most sense to visualize along with the phenotypic links.

The 3D arc plot is interactive. One can select individual arcs to get further information on the genes involved in the specific connection, along with gene ontology information, if it has been loaded (Figure 1A). Furthermore, one can filter the dataset for genes of interest, such that only connections that involve these genes are displayed.

#### The heat map representation

The heat maps of gene-associated values for each phenotype are generated and displayed separately for each time point. The rows and columns are clustered using agglomerative hierarchical clustering. The user can choose between single, average or complete linkage and Euclidean or Manhattan distance.

#### The line plot representation

The line plots are timelines showing the evolution of gene-associated values over time for every phenotype. The global view depicts the plots for the entire dataset. Similar to the heat map view, a mouse hovering event will pop-up a new window for the chosen gene for closer analysis.

#### Networks and data integration

To get insights into the functionality and properties of the genes associated to phenotypic changes, gene ontology (GO), protein–protein interaction, metabolic and other types of networks can be loaded from a file or from the STRING database [21,22] and visualized dynamically. Nodes in the network are dynamically highlighted at each time point to reflect correspondence to genes underlying the particular phenotypic connections at the respective time point. A network of genes can also be automatically generated from the data, where the links indicate that two genes are involved in the same phenotype. This network is recalculated and updated for each time point. For performance and display reasons, only networks with less than 500 nodes will be generated and shown. To optimize the drawing space, we display the networks using a force-directed layout. The GO term networks use this non-classical layout too, as we believe the focus should be on the individual highlighted terms rather than on how they are connected. Nevertheless, the networks are interactive, so the layout can be rearranged by the user. Other network layout options are planned for future implementation.

Additionally, right-clicking a gene/protein name within the network will provide links to several databases: UniProt [23], Ensembl [24], Entrez Gene [25], Entrez Proteins [26] and KEGG [27]. The query results of searching for this entity in any of these databases will be opened and displayed in a browser.

#### Other considerations

The tool is interactive, with zoom-in, zoom-out, pan and rotate capabilities. Filters for specific genes or phenotypes can be set. There are several color schemes available for use (from http://colorbrewer2.org/), including color-blind schemes or single color display.

The input consists of a special format space-delimited file, as described in Table S2. Considering the heterogeneity of possible input data, a normalization option is not available and should be performed beforehand if needed. Network files (GO or other types of networks) can be loaded in the format described in Table S3. The data employed in the results presented in this paper is available on the website at http://phenotimer.org/samplefiles.html. These files can be loaded directly into PhenoTimer, but, in order to obtain the same figures, thresholds must be applied to the phenotypic outcomes as described for each dataset.

More details on the workflow and functionality of the tool can be found in Figures S2 and S3, and on the website at http://phenotimer.org/tutorial.html.

### Limitations

PhenoTimer performs best on Mac OSX and Windows. Compatibility issues of Processing in Linux may impair the performance of PhenoTimer in this environment. For reasons of CPU load and physical visualization limits, it is recommended not to visualize datasets that exceed the following dimensions: a few thousand genes x 50 phenotypes x 100 time points. The main memory usage limitation is the number of phenotypes.

## Results and Discussion

Visualization of large-scale datasets is crucial for the understanding of key regulatory factors and a better elucidation of biological processes. Time-resolved data adds an extra challenge that tools currently available to biologists hardly meet. In this section, we demonstrate how to use PhenoTimer on time-resolved multiple-phenotype data for quick pattern identification and generation of new hypotheses.

### Discovering patterns in cell cycle regulation

One of the best examples of highly time-regulated processes is the cell cycle. Intensely studied, it still poses interesting questions for biologists, with implications in senescence processes and disease [28].

#### Progression dynamics of cell division defects

We illustrate an investigation of phenotypic patterns throughout the cell cycle as they arise from perturbations in the system. The data comes from a whole-genome siRNA knockdown study on genes essential for cell division, as described in [29]. Here, they suppress the function of one selected gene at a time and follow the cell division process within cell populations. The genes essential to the cell cycle will cause cell division defects upon knockdown. These defects fall into seven main categories, based on morphological feature scoring: “mitotic delay”, “binuclear”, “polylobed”, “grape”, “large”, “dynamic” and “apoptosis”. The cells transition through different phenotypic stages before dying or becoming arrested into a particular morphology. A succession of phenotypes therefore characterizes the imaged cell populations.

We use PhenoTimer to visualize how the cell populations transition from one phenotype to the other upon knockdown of selected genes (Figure 1).

We only represent transitions to the most prominent phenotypes at every time point (i.e. maximally scored for the respective gene knockdown). We apply thresholds to the phenotypic scores assigned to every suppression event according to the values mentioned in [29].

We can easily observe patterns, as shown in Figure 1. Transitions to the “polylobed” (purple) and “apoptosis” (green) phenotypes are prominent features that arise. “Polylobed” is a widely recurring morphology that occurs mostly after the “mitotic delay”, “dynamic” or “binuclear” phenotypes. In fact, one notices a relatively stable alternation between the “binuclear” and “polylobed” phenotypes. Both constitute cytokinesis defects and are likely to succeed each other. The several transitions to apoptosis are expected, since many of the knockdowns will perturb the cellular system strongly enough to kill the cell.

Besides these, one can also identify less common transition patterns: “mitotic delay” to “dynamic” at large time intervals, fairly uniformly distributed throughout the time course; “mitotic delay” to “large”, more frequent towards the end; “mitotic delay” to “grape”; or “grape” to “large”. By comparing these, we can quickly identify the more prevalent (polylobed, apoptosis, binuclear) and the rarer phenotypes (grape, large). We also get an overview of the timing in the cell cycle when a particular transition can occur. The rarer transitions occur at longer time intervals, indicating that they are more likely slow and final transitions, as opposed to the transitions to polylobed and apoptosis that are more homogeneous throughout the time course and thus more frequent and faster (see Figure S4 for single phenotype plots).

By visualizing the GO network dynamically along the time course, one can look at the genetic background that explains the phenotypes. In this way, the user can understand what kind of functional roles of genes/proteins would lead to certain phenotypic patterns and how they evolve with time. The timing of the phenotypic transitions driven by gene knockdown events reveals a succession of molecular functions linked to the cell cycle (box in Figure 1). The enriched functionality is summarized for the entire time course in Figure S5. Periodic spikes can be observed for some cell division-, complex assembly- and metabolic-linked processes. The reconstructed functionality timeline reflects, to some extent, the expected chronology of events throughout the cell cycle, e.g. spindle assembly-related events appear earlier in the time course compared to events involving genes with roles in the ubiquitination pathway. This suggests the possibility of a link between the timing of protein activity within the cell cycle and the timing of the phenotypic onset upon this protein’s deregulation.

By identifying the genes that determine the same phenotypic transitions, one can even infer new functions for unknown genes. For instance, gene *MGC13053* in Figure 1A is of unknown function, but is involved in the same type of transition as genes *PLCB2*, *SPATC1* and *PKN3*. These participate in ribonucleotide binding processes, e.g. tubulin binding, centrosomal activity, phosphorylation events (according to GeneCards [30]). Thus, a valid hypothesis to test might be whether *MGC13053* affects microtubule nucleation dynamics. As such, it could be involved in defective spindle pole assembly or chromosome segregation, resulting in cytokinesis arrest, as the phenotype suggests.

From the phenotypic transition profiles of the cells, we were able to infer a network of genes that may be synchronously transcribed or have products involved in the same pathway. We hypothesize that those genes whose knockdown causes the same succession of phenotypes in a synchronized manner should be involved in closely related processes, at least temporally if not also spatially. 482 such genes were found. The resulting networks are depicted in Figure S6. The genes involved in these hypothesized synchronous events have as first phenotype upon silencing either “binuclear”, “polylobed”, “apoptosis” or “dynamic”. 62.4% of the interactions have been validated with GeneMania [31,32]. The respective genes were mostly involved in mRNA splicing, their products likely constituting parts of the spliceosome (see Table S4). Examples for four of the largest gene network modules are shown in Figure S7. The rest of the interactions are novel and should be tested experimentally.

K-means clustering of these genes according to their phenotypic succession profiles places them in four classes, similar to the classification according to the first resulting phenotype (Figure S8).

These genes are probably involved in critical points of later stages of cell division, which are complex and require good coordination. As such, some of the novel interactions identified may be of significant interest.

#### Disease connection discovery

In this section, we show how PhenoTimer could facilitate the discovery of potential links between diseases. We investigated the impact of peak transcription events throughout the cell cycle on different types of cancer. For this, we used transcription profiles of 600 essential genes that periodically fire throughout the progression of the cell cycle [33]. This means that the peak of transcription for these genes always occurs at the same time point. We map the transcription peaks only for the genes enriched in cancer pathways, as obtained from bioCompendium (http://biocompendium.org/).

**Figure 2:**
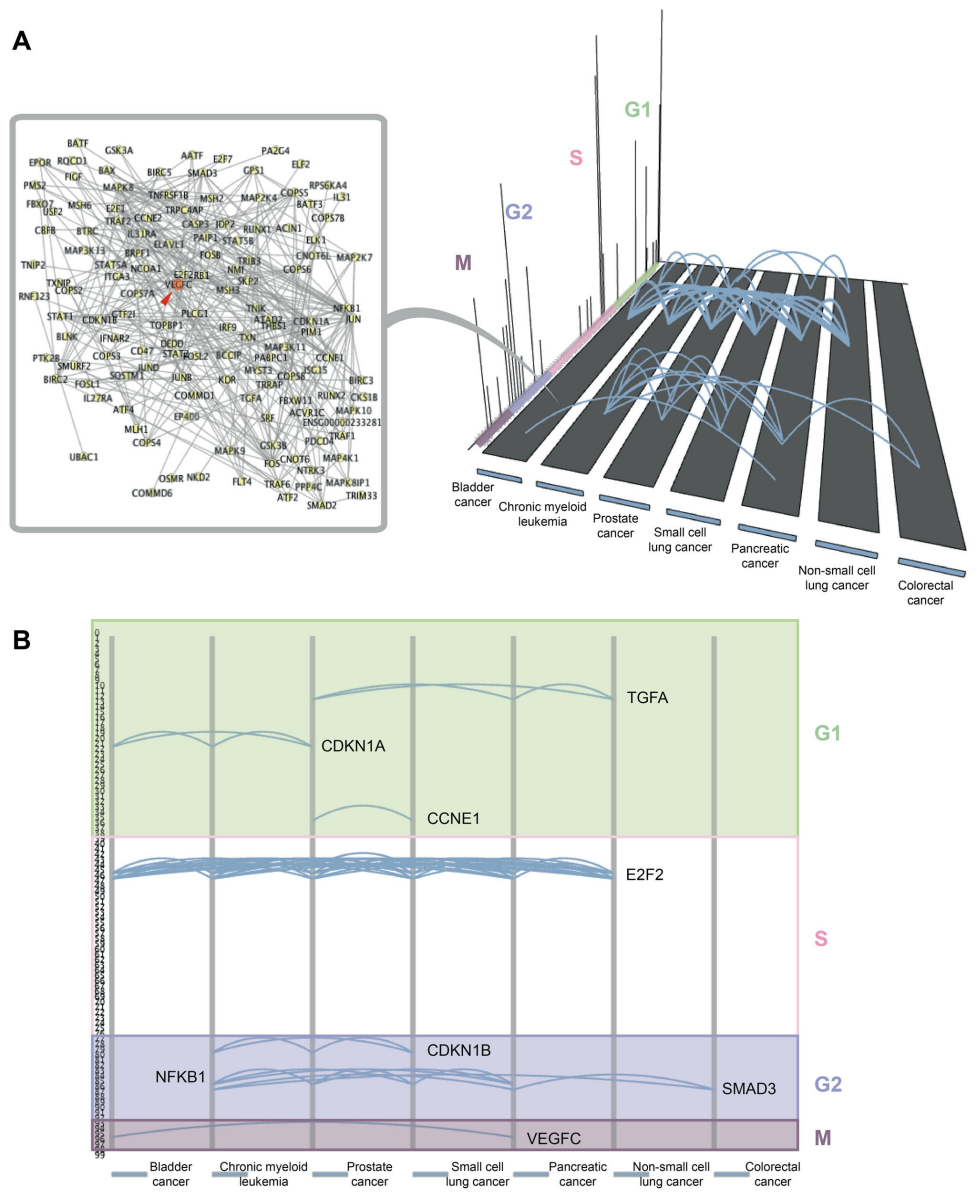
Transcription events linking cancer pathways. (A) Peaks of transcriptional activity for periodic genes within the cell cycle are shown throughout the cell cycle phases, along with the cancer pathways in which they are enriched. Two cancer pathways are connected if they share at least one enriched gene at the particular time point. The network neighborhood of the genes involved in the connections is also shown, as retrieved from STRING. Gene *VEGFC* is highlighted in red. (B) The genes shared by several cancer pathways are highlighted through the course of the cell cycle.

Figure 2 illustrates cancer pathways that share at least one enriched gene that has a periodic peak of transcription at a certain moment in the cell cycle. Some highly active transcription events, like those in the beginning of S phase or middle of G2 phase, are common to almost all types of cancers. In contrast, some of the others, especially in the M-phase, prove to be rarer, referring to a gene that is enriched in only two cancer types (bladder and pancreatic cancer). The respective gene is *VEGFC* [ENSG00000150630], a growth factor active in angiogenesis and endothelial cell growth. The network it is involved in, derived from STRING, is also shown in Figure 2A. Figure S9 shows how *VEGFC* relates to all the other periodic genes enriched in at least one pathway disrupted in cancer. It mostly connects through genetic interactions or co-expression to the rest of the network and is commonly enriched in cancer pathways with the directly linked partners, *E2F2* and *NFκB1*, both peaking after the G1 phase. It is possible that the disruption of the links with *VEGFC* upon malfunctioning of either one or the other of these two proteins plays a role as tumor-triggering factor. This analysis indicates that the regulation of most cancers might involve very similar mechanisms for replication of genetic material, but the errors of cell division leading to disease may be cancer type-specific.

This example of mapping transcription events to pathways involved in cancer provides an idea of how PhenoTimer might be used for similar studies. While this model was rather naive, we are confident of the potential of this tool to provide new insights about common mechanisms for disease regulation and progression, given a more complex context.

### Linking drug abuse phenotypes

Drugs of abuse act on the brain reward system and employ similar mechanisms to generate addiction. The impact on human health makes this an intense topic of research. Elucidating the genes and pathways commonly affected by several drugs can help us better understand the downstream effects of drug intake, as well as identify potential side effects of drug combinations.

We looked at transcriptome alterations in the mouse striatum upon acute administration of six addictive drugs: nicotine, ethanol, cocaine, methamphetamine, heroin and morphine. We used detailed time course profiles of gene expression as described in [34] to analyze common influences of pairs of drugs on different gene classes. Transcription levels are measured at intervals of 1, 2, 4 and 8 hours after drug injection.

From the set of 42 genes identified as drug-responsive in the paper, we looked at how the genes with transcription values in the lower and upper quartiles are commonly regulated by pairs of drugs. We term these genes as relatively “lowly” or “highly expressed” within the group. Table S5 documents the thresholds applied for the classification. We use PhenoTimer to plot connections between drug mechanisms through time.

**Figure 3:**
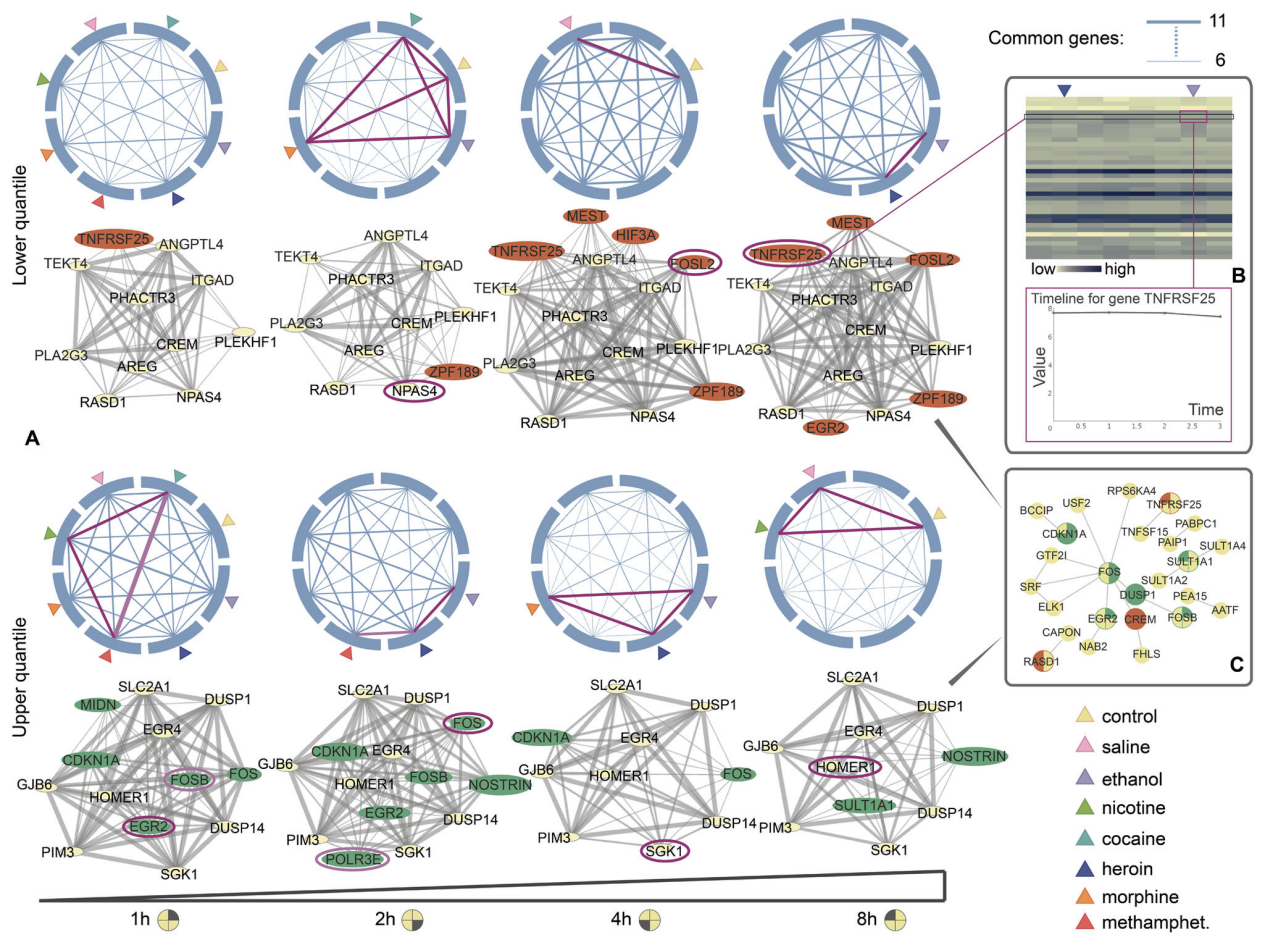
Similar mechanisms of drug-induced gene regulation for up to 8 hours after treatment. (A) Pair-wise connections between drugs and networks of genes affected by the same drug are visualized using PhenoTimer for every time point. Two drugs are connected if they similarly regulate the same gene. Two genes are connected in the network if they respond to the same drug(s). Thickness of links corresponds to the number of genes, or drugs, respectively, shared by two partners. Only genes with transcription values in the lower quartile (top) or upper quartile (bottom) are taken into account. The core gene network is depicted in yellow and the variable gene elements (i.e. that don’t appear at every time point) are highlighted in orange (lower quartile) and green (upper quartile). Links between drugs are depicted in magenta and pink if they contain the highest number of commonly regulated genes. The genes specific for that particular link only are circled in the same color in the network below. (B) Heat map of the gene expression values after 8 hours of drug induction is shown for every drug. The line corresponding to gene *Tnfrsf25* is highlighted and the columns corresponding to ethanol and heroin are also indicated. The graphic of transcription counts throughout the time course for this gene after heroin induction is shown below. Both images have been generated using PhenoTimer. (C) The network of human homologs for the relatively lowly and highly expressed genes. The variable genes are highlighted in orange (lower quartile) and green (upper quartile), along with the time points when their values are in the required quartile range.

Figure 3A shows drugs that have similar impact on the transcription of genes at every time point. If the expression of a gene is in the lower (respectively, upper) quartile upon injection of both drugs A and B at a particular time point, we connect the two drugs. The thickness of the line corresponds to the number of genes that are commonly lowly or highly expressed at that time point between both drug treatments. In parallel to pairwise evolution of drug connections through time, we show the networks of genes that are similarly affected by the same drug(s). The networks are generated automatically from the data using PhenoTimer. Thus, the figure shows how connections evolve in time between 1) drugs that affect the same genes and 2) genes affected by the same drugs. We only take into account relatively lowly and highly expressed genes in both cases.

The first observation is that mechanisms of action are very similar among drugs of abuse. For the genes whose expression is in the lower quartile, we notice an increase with time in the number of commonly affected genes by pairs of drugs. An inverse relation is observed for the upper quartile genes, as there are more of these genes influenced by the same two drugs in the beginning compared to the end of the time course. This suggests that drugs of abuse impact many of the differentially expressed genes by lowering their expression rather than enhancing it.

The gene networks allow us to identify the stable and the variable elements involved in the drug connections. At every time point, a connection between two genes means that their expression is influenced by the same drug(s). The thickness of the link corresponds to the number of common drug influences. The yellow nodes form the core gene network that stays constant throughout the time course. Among the lowly expressed genes, many core components were related to proliferation; the highly expressed genes, on the other hand, were preponderantly involved in phosphorylation-mediated signalling and transcription events (Table S6). This suggests that common and consistent effects of drug abuse include reduction of cell proliferation and alteration of transcription and signalling pathways as a response to stress.

The orange (lower quartile) and green (upper quartile) nodes are variable elements, i.e. the genes that appear and disappear in the network at different time points. These include major regulators of transcription and cell division, as well as genes involved in hypoxia response (see Table S6). The variability in the transcriptional response of these genes might be explained by the fact that they tend to be more robust to perturbations.

Figure 3C displays the human homolog network extracted from STRING for the entire set of drug-responsive genes. The lowly and highly expressed genes that are commonly regulated by drugs are highlighted in the corresponding colors and the time points of common regulation are depicted. More of the core network genes tend to be conserved in human compared to the variable genes.

The gene networks are quite dynamic through time, underlying small differences in the mechanisms of action of different drugs. In fact, despite the similarities between drug connections, their evolution is dynamic as well. The drug links colored in magenta and pink highlight the pairs of drugs that act on the highest number of common genes and have at least one specific gene for the connection (i.e. unique, not encountered in the other connections). The gene specific only for that pair of drugs at a time point is circled in the same color in the network below. For the genes in the lower quartile, we notice that gene *Tnfrsf25*, a member of the tumor necrosis factor receptor superfamily [ENSG00000215788], is uniquely downregulated only by ethanol and heroin after 8 hours. Heat map and line plot analysis for this gene shows that the downregulation is a very slight effect and would have been difficult to capture otherwise (Figure 3B). The two drugs also uniquely affect genes *Fos* and *Sgk1*, whose transcription values are in the upper quartile range after 2 and 4 hours, respectively. *Fos* is a regulator of cell proliferation and differentiation and has been associated with apoptotic cell death [35], while *Sgk1* is a serine/threonine-protein kinase with important roles in cellular stress response [36]. This suggests there may be some stronger crosstalk between ethanol- and heroin-regulated pathways, especially those of neuronal death and tumor development. Hence, the effects of ethanol consumption might resemble more those of heroin than of the other drugs.

Other specific genes include *Fosb* for cocaine and methamphetamine (both psychostimulants), which share effects on *Egr2* also with nicotine; and *Polr3e* for ethanol and methamphetamine. Morphine has the same effects on *Sgk1* as ethanol or heroin after 4 hours.

Differences between drug effects are clearer if we look at the time evolution of drug and gene connections for stimulants (cocaine, methamphetamine) and depressants (heroin, morphine), as depicted in Figure S10. We observe an accumulation of lowly expressed genes, as well as reduction of highly expressed genes with time between all pairs of drugs. Surprisingly, among the upper quartile genes, there are more commonly affected genes for the pairs cocaine-heroin and morphine-methamphetamine, even though they link different drug classes. In this case, the last gene network depicts the pairs of genes that are most highly and constantly affected by the same drug, the other variable elements seeming to lower their expression with time. More and more genes become relatively lowly expressed, perhaps a consequence of enhancement of transcription factor control.

The 42 true positive genes found in the study were classified in the paper into four subclasses according to their gene expression patterns: A (involved in behavioral sensitization and reward learning), B1 (reward learning and drug dependence), B2 (drug dependence) and B3 (anti neurotoxic response) [34]. We look at lowly expressed genes in these subclasses and how they are commonly affected by pairs of drugs. Figure S11 depicts an even more dynamic connection landscape for these subclasses, in particular for those belonging to group B1. Groups A and B3 display contrasting regulation methods: drugs regulate more genes similarly in the beginning of the treatment for group A and towards the end of the treatment for group B3. Connections in group B2 are rather constant. The genes highlighted in red are affected by pairs of drugs in similar ways. We notice that, in most cases, the products of these genes are involved in pathways where the interaction partners are not affected in a similar manner by the drugs. This suggests that some compensatory mechanism might arise to account for these localized alterations of protein content. This should be further investigated.

The similarities identified in the action of different drugs reveal an uneven pattern of regulation within a single drug class or a gene group, and several similarities between different classes. This further expands on the complexity of addiction mechanisms. This example also highlights the potential of using PhenoTimer to identify synergistic effects of drugs, which could have implications for designing drug therapies.

## Conclusions

We have shown how PhenoTimer can help with the better understanding of phenotypic relations by connecting back to the genetic background and by embedding time information. The tool has proven to be useful in fast patterning of phenotypic transition profiles within the cell cycle upon perturbation, as well as in the identification of similarities in drug action and potential novel links between diseases.

Compared to similar software for time-resolved data, PhenoTimer introduces several new features. To our knowledge, no other tool is currently available for specifically mapping connections between phenotypes in the form of arc projections. Furthermore, these relationships and their genetic determinants can be tracked dynamically, along with network and functionality information. The alternation between different 2D and 3D views allows a detailed inspection of the data, and global patterns can be easily detected. In short, PhenoTimer enables an interactive exploration of multidimensional phenotypic screens for global trends and single time point details, in an adaptable manner that allows the integration of dynamic network information.

We anticipate that the use of this tool is not limited to the examples we have shown, but its features are suited to a whole range of biological data. In general, it is relevant for the analysis of interesting subsets of high-throughput expression or imaging screens, as well as other experiments. As application, PhenoTimer could also be used for the visualization and analysis of spatiotemporal programs encoded within and among chromosomes. Identifying common points in the progression of various diseases could provide new strategies for combined treatment and drug repurposing. Visualization of common variations among bacterial populations in the human gut through time would give us further insight into the dynamics of the microbiota of the gut. Additionally, it can be used for quality control of different replicates in an experiment or for comparison between different tissues. We thus envision phenotypic patterning of processes from the smallest to the largest scale, for a global view of time-dependent regulation and rhythms governing life.

## Supporting Information

Figure S1 **The different visualization modes in PhenoTimer.**
(A) 3D arc view. Connections between phenotypes are represented as arcs, the color encoding directionality (in case it is not needed, single color depictions can be used). The height of the arc corresponds to the number of genes involved in the connection. Bar charts with values for every time point can be loaded as well. (B) 2D arc view. Connections are represented as 2-dimensional arcs, with the same color coding as in (A). The thickness of the arc corresponds in this case to the number of genes. (C) Circular view. The connections between phenotypes are visualized in a circular manner for every time point. (D) Heat map view. The gene-associated values are visualized for each phenotype in a separate heat map for every time point. The user can choose the clustering method. The heat maps are expanded upon hovering and can be individually analyzed. (E) Line plot view. The gene-associated values are visualized as timeline plots for every phenotype. The graphs are expanded upon mouse hovering.
(TIF)

Figure S2 **Details of PhenoTimer graphical user interface.**
(A) Part of the canvas where the different 2D/3D graphical representations are drawn. (B) Part of the canvas where the different 2D network representations are drawn. (C) Controls for setting thresholds for phenotypic values. One can set new value ranges by dragging the sliders. (D) Slider controller for moving through time. Pressing the key “t” allows switching between visualizing connections for a single time point and for all time points up to the current one. (E) Slider that allows setting the time interval for arc display. (F) Controller for changing the unit height (in 3D) or width (in 2D) of the arcs, for better emphasis of visualized data. (G) Slider that allows the changing of the arc transparency, for optimized visualization (default is 20%). (H) Pop-up that indicates the action that can be taken using the corresponding slider.
(TIF)

Figure S3 **PhenoTimer workflow.**
The experimental data coming from medium or high-throughput gene expression or imaging screens for which time-lapse recordings have been made is formatted into a special input file similar to the one in the top panel, parsable by PhenoTimer. This file is then loaded into PhenoTimer for processing. The tool produces already at this point the visual output, but one might wish to first set thresholds for gene-associated values for each phenotype, otherwise all phenotypes might appear connected. After this step, one is ready to visualize the data in different view modes and integrate network information (bottom panel).
(TIF)

Figure S4 **Single phenotype transition plots, as produced by PhenoTimer.**
Each plot visualizes only transitions to and from phenotype “polylobed” (A), “apoptosis” (B), “grape” (C) and “large” (D), respectively. Prevalent phenotypes (A and B) are clearly distinguishable from rarer ones (C and D). This holds even when considering only transitions towards the phenotype of interest, depicted in purple (polylobed), green (apoptosis), blue (grape) or red (large).
(TIF)

Figure S5 **Timeline of molecular functions enriched for genes essential for cell division.**
The gradient highlights the number of genes whose silencing causes transitions at a particular time point and that are enriched for the respective molecular function. The plot was produced in R.
(TIF)

Figure S6 **The hypothesized network of synchronously activated genes or proteins involved in the same pathway.**
The nodes correspond to silenced genes and the genes are connected if they show the exact same phenotypic succession events upon knockdown. The genes are colored according to the first phenotype shown in the cells upon knockdown. Out of all interactions hypothesized, 62.4% have been validated from the literature using GeneMania, with the following distribution: co-expression 64.24%, physical interactions 14.68%, genetic interactions 11.16%, co-localization 5.46%, predicted 4.37%, shared protein domains 0.09%. The networks were visualized using Cytoscape.
(TIF)

Figure S7 **Connections from the literature between genes of four hypothesized interactive modules.**
The cells where these genes are knocked down adopt a binuclear phenotype after: (A) 16.5 hours; (B) 15 hours; (C) 15.5 hours; (D) 26 hours. The networks were retrieved from GeneMania.
(TIF)

Figure S8 **K-means clustering reveals 4 clusters of genes with similar phenotypic succession profiles.**
The clustering for the first two principal components is displayed. The clustering was performed on the vectors of phenotypic assignment of most prevalent phenotype at each time point for every gene. The clustering and plotting were performed in R.
(TIF)

Figure S9 **The network of genes affected in cancer that are also periodically transcribed throughout the cell cycle.**
The genes that related to more than one type of cancer are highlighted. Circle colors indicate the different cancer types where genes are enriched. Genes highlighted in this way are the ones involved in the connections visualized using PhenoTimer. In general, genes affected in the same cancer types interact physically or genetically. The network was retrieved from GeneMania and further edited in Cytoscape.
(TIF)

Figure S10 **Mechanistic similarities of stimulants (cocaine, methamphetamine) and depressants (heroin, morphine).**
Action upon genes with transcription profiles in the lower quartile (top) and upper quartile (bottom) ranges is depicted. Two drugs are connected if treatment with either of them results in similar levels of gene expression for at least one gene. The thickness (2D) or height (3D) of the arcs corresponds to the number of genes commonly affected by two drugs. The networks connect genes that respond to the same drug(s). The thickness of the edges corresponds to the number of drugs to which the pair of genes is responsive. Orange nodes (top) and green nodes (bottom) are variable elements, while yellow nodes correspond to the core gene network that stays the same throughout the time course. The plots have been obtained using PhenoTimer and then combined and annotated to emphasize different aspects of the analysis.
(TIF)

Figure S11 **Drug similarities in action on different gene subclasses.**
Two drugs are connected if they act similarly on at least one common gene, the thickness of the links indicating how many genes are influenced by that pair of drugs. To the right, the networks of the genes corresponding to the different subclasses have been retrieved from GeneMania. The genes highlighted in red appear in drug pair connections. The circular plots have been obtained individually using PhenoTimer and then combined and annotated to emphasize different aspects of the analysis.
(TIF)

Table S1 **Comparison of the different view modes of PhenoTimer.**
The table lists the comparative strengths and weaknesses of the different graphical representations used in PhenoTimer.
(DOC)

Table S2 **Example of an input file loadable into PhenoTimer.**
The first column specifies the gene names, the second column the phenotypes and the subsequent columns list the gene-associated values at each time point. The fields must be separated by white space.
(DOC)

Table S3 **Example of network files loadable into PhenoTimer.**
(a) Input file containing the GO enrichment specifications: the columns must specify the GO identifiers, the corresponding descriptions, the p-values of the enrichment and the genes that are enriched for each category, separated by “|”. (b) Along with the enrichment file, an interaction file should also be loaded into PhenoTimer, specifying the interaction partners in the network, one pair per line. The format is the same for other types of networks (e.g. PPIs, metabolic etc.). All these are tab-separated fields.
(DOC)

Table S4 **GO enrichment for the network of hypothesized synchronous genes.**
The table lists the molecular functions of all the genes in the mitotic progression dataset whose knockdown causes identical phenotypic successions to at least one other gene.
(DOC)

Table S5 **Quartile calculations for the measured transcriptional levels upon drug intake.**
The table lists the quartiles of the normalized and log_2_-transformed mRNA abundance measured for each drug treatment. The lower (25%) and upper (75%) quartile values are used as thresholds for subsequent visualization and analysis.
(DOC)

Table S6 **The functionality description of core network and variable genes similarly regulated by drugs.**
Lowly and highly expressed genes are defined as before. The descriptions were taken from UniProt.
(DOC)
