# Supplementary figures and images for: PhenoTimer: Software for the Visual Mapping of Time-Resolved Phenotypic Landscapes
Source: PLoS One. 2013 Aug 12;8(8):e72361. doi: 10.1371/journal.pone.0072361 (PMC3741141; doi:10.1371/journal.pone.0072361)

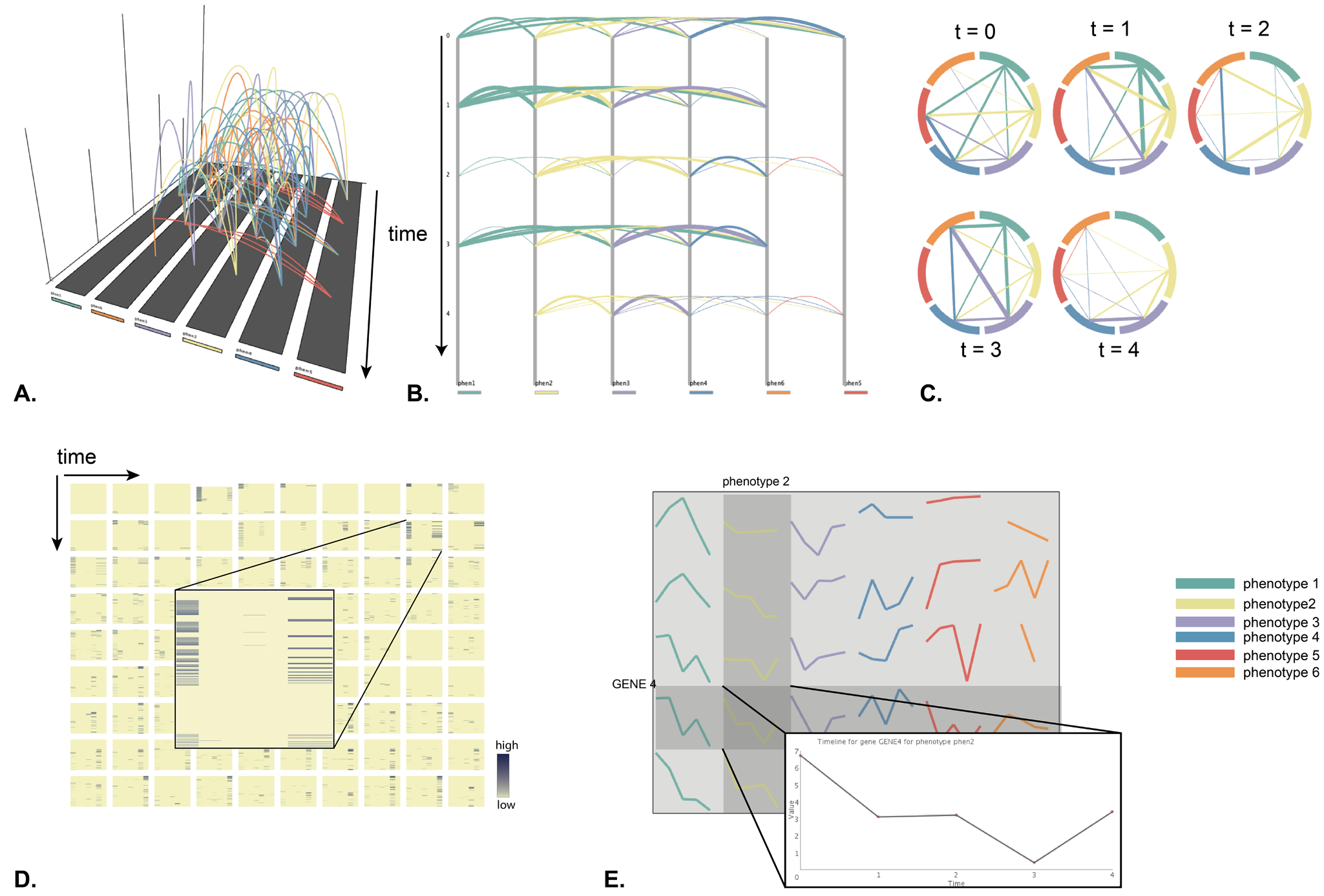

Supplement: Figure S1 — The different visualization modes in PhenoTimer. (A) 3D arc view. Connections between phenotypes are represented as arcs, the color encoding directionality (in case it is not needed, single color depictions can be used). The height of the arc corresponds to the number of genes involved in the connection. Bar charts with values for every time point can be loaded as well. (B) 2D arc view. Connections are represented as 2-dimensional arcs, with the same color coding as in (A). The thickness of the arc corresponds in this case to the number of genes. (C) Circular view. The connections between phenotypes are visualized in a circular manner for every time point. (D) Heat map view. The gene-associated values are visualized for each phenotype in a separate heat map for every time point. The user can choose the clustering method. The heat maps are expanded upon hovering and can be individually analyzed. (E) Line plot view. The gene-associated values are visualized as timeline plots for every phenotype. The graphs are expanded upon mouse hovering. (TIF) [file pone.0072361.s001.tif]

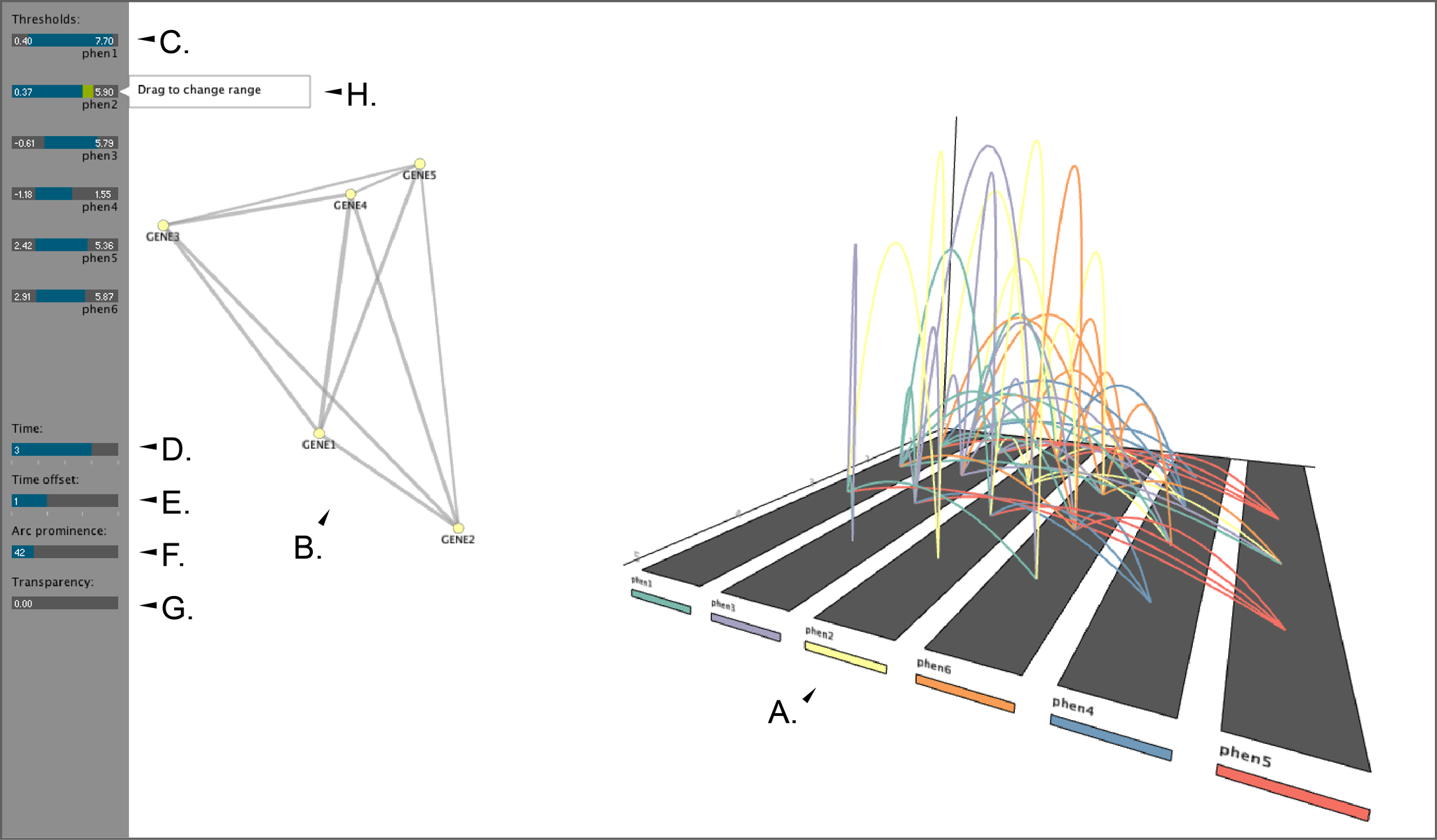

Supplement: Figure S2 — Details of PhenoTimer graphical user interface. (A) Part of the canvas where the different 2D/3D graphical representations are drawn. (B) Part of the canvas where the different 2D network representations are drawn. (C) Controls for setting thresholds for phenotypic values. One can set new value ranges by dragging the sliders. (D) Slider controller for moving through time. Pressing the key “t” allows switching between visualizing connections for a single time point and for all time points up to the current one. (E) Slider that allows setting the time interval for arc display. (F) Controller for changing the unit height (in 3D) or width (in 2D) of the arcs, for better emphasis of visualized data. (G) Slider that allows the changing of the arc transparency, for optimized visualization (default is 20%). (H) Pop-up that indicates the action that can be taken using the corresponding slider. (TIF) [file pone.0072361.s002.tif]

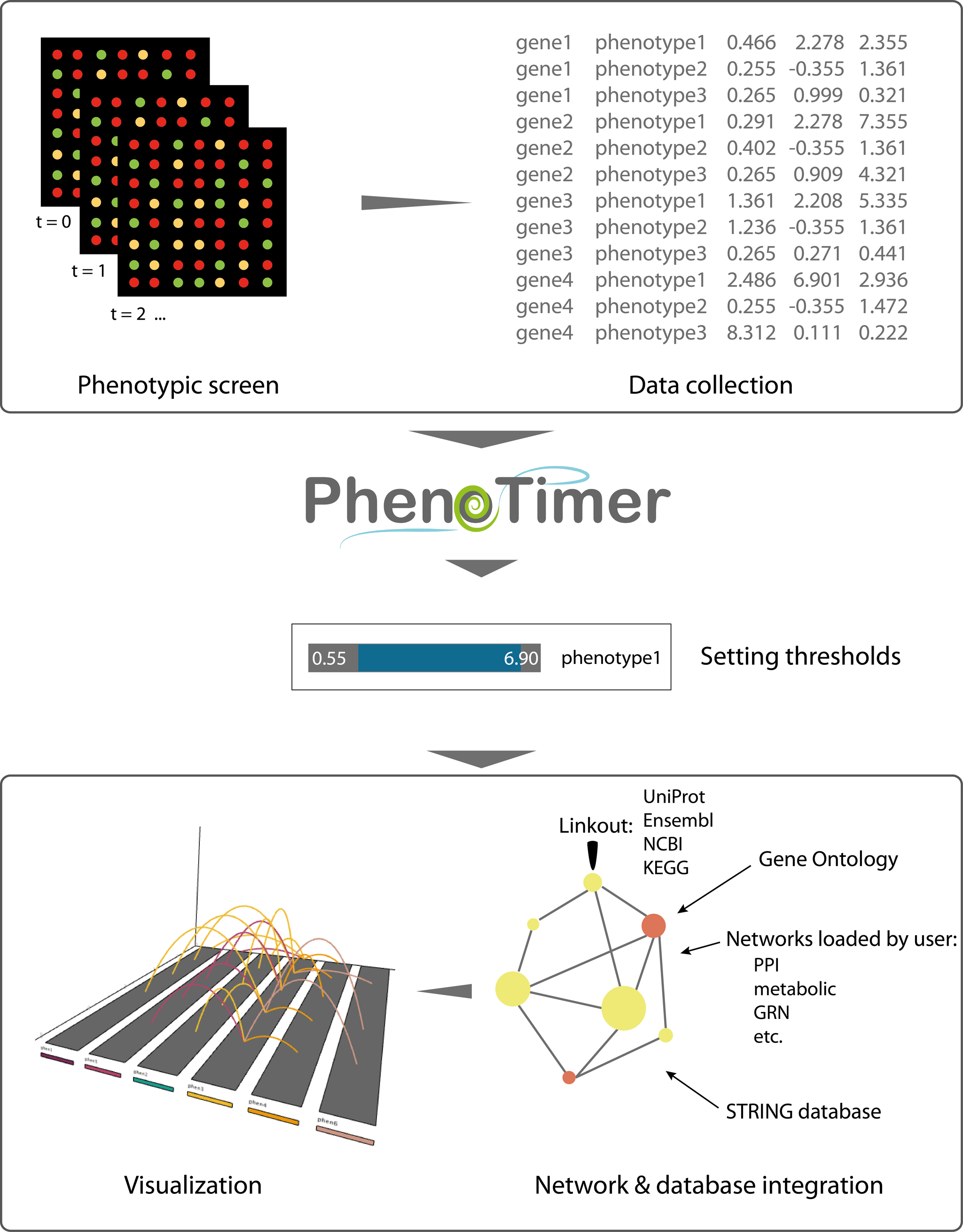

Supplement: Figure S3 — PhenoTimer workflow. The experimental data coming from medium or high-throughput gene expression or imaging screens for which time-lapse recordings have been made is formatted into a special input file similar to the one in the top panel, parsable by PhenoTimer. This file is then loaded into PhenoTimer for processing. The tool produces already at this point the visual output, but one might wish to first set thresholds for gene-associated values for each phenotype, otherwise all phenotypes might appear connected. After this step, one is ready to visualize the data in different view modes and integrate network information (bottom panel). (TIF) [file pone.0072361.s003.tif]

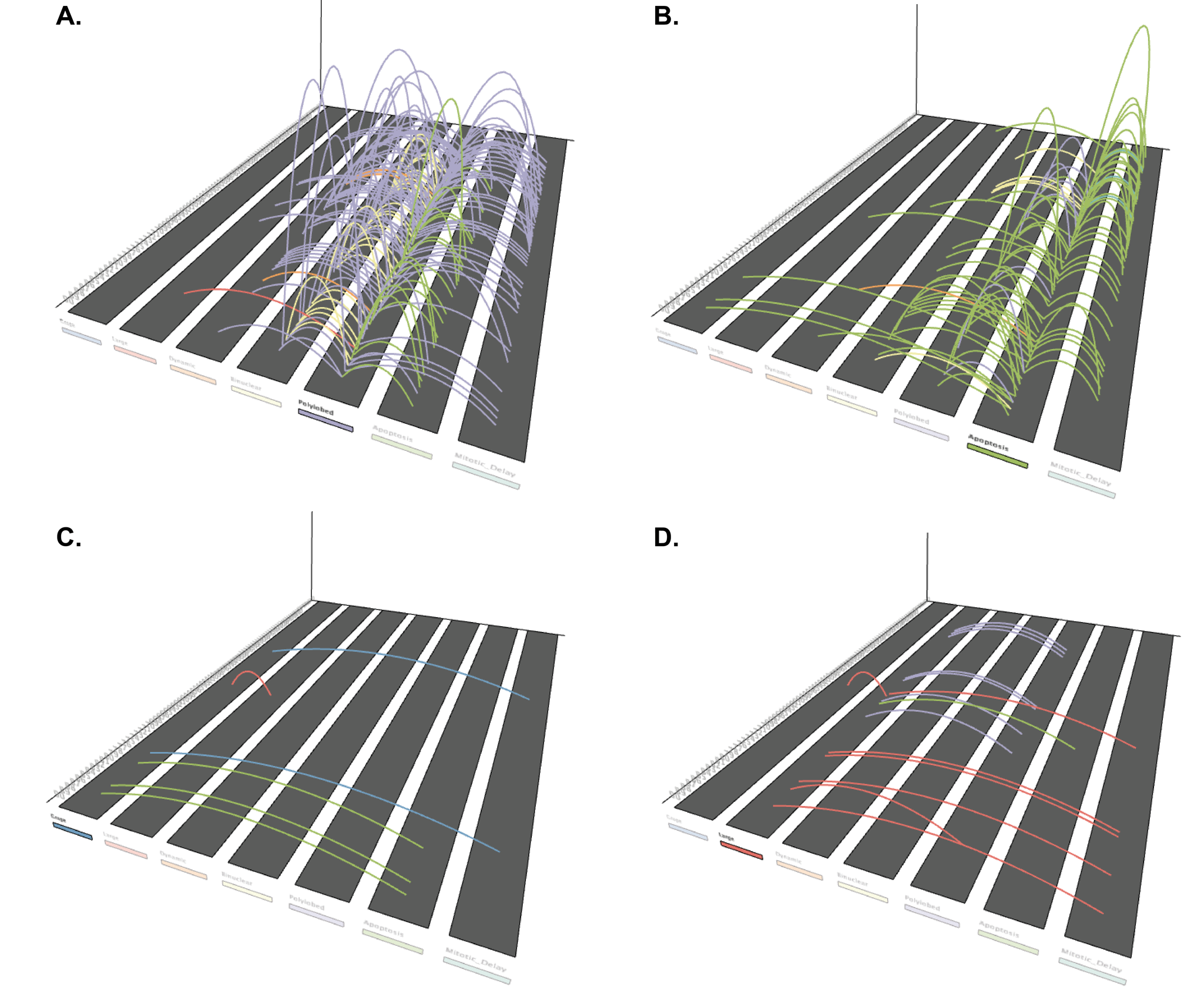

Supplement: Figure S4 — Single phenotype transition plots, as produced by PhenoTimer. Each plot visualizes only transitions to and from phenotype “polylobed” (A), “apoptosis” (B), “grape” (C) and “large” (D), respectively. Prevalent phenotypes (A and B) are clearly distinguishable from rarer ones (C and D). This holds even when considering only transitions towards the phenotype of interest, depicted in purple (polylobed), green (apoptosis), blue (grape) or red (large). (TIF) [file pone.0072361.s004.tif]

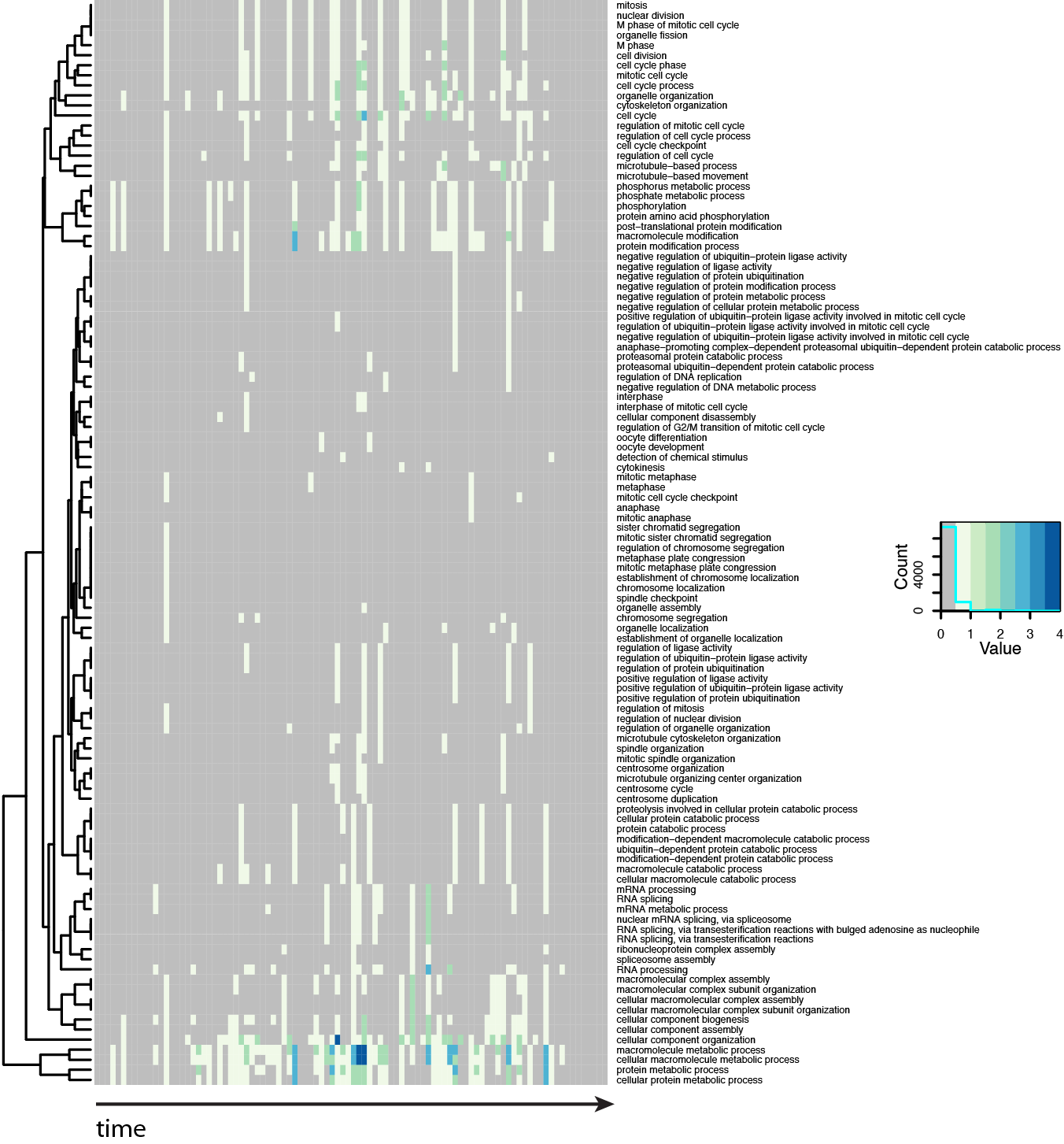

Supplement: Figure S5 — Timeline of molecular functions enriched for genes essential for cell division. The gradient highlights the number of genes whose silencing causes transitions at a particular time point and that are enriched for the respective molecular function. The plot was produced in R. (TIF) [file pone.0072361.s005.tif]

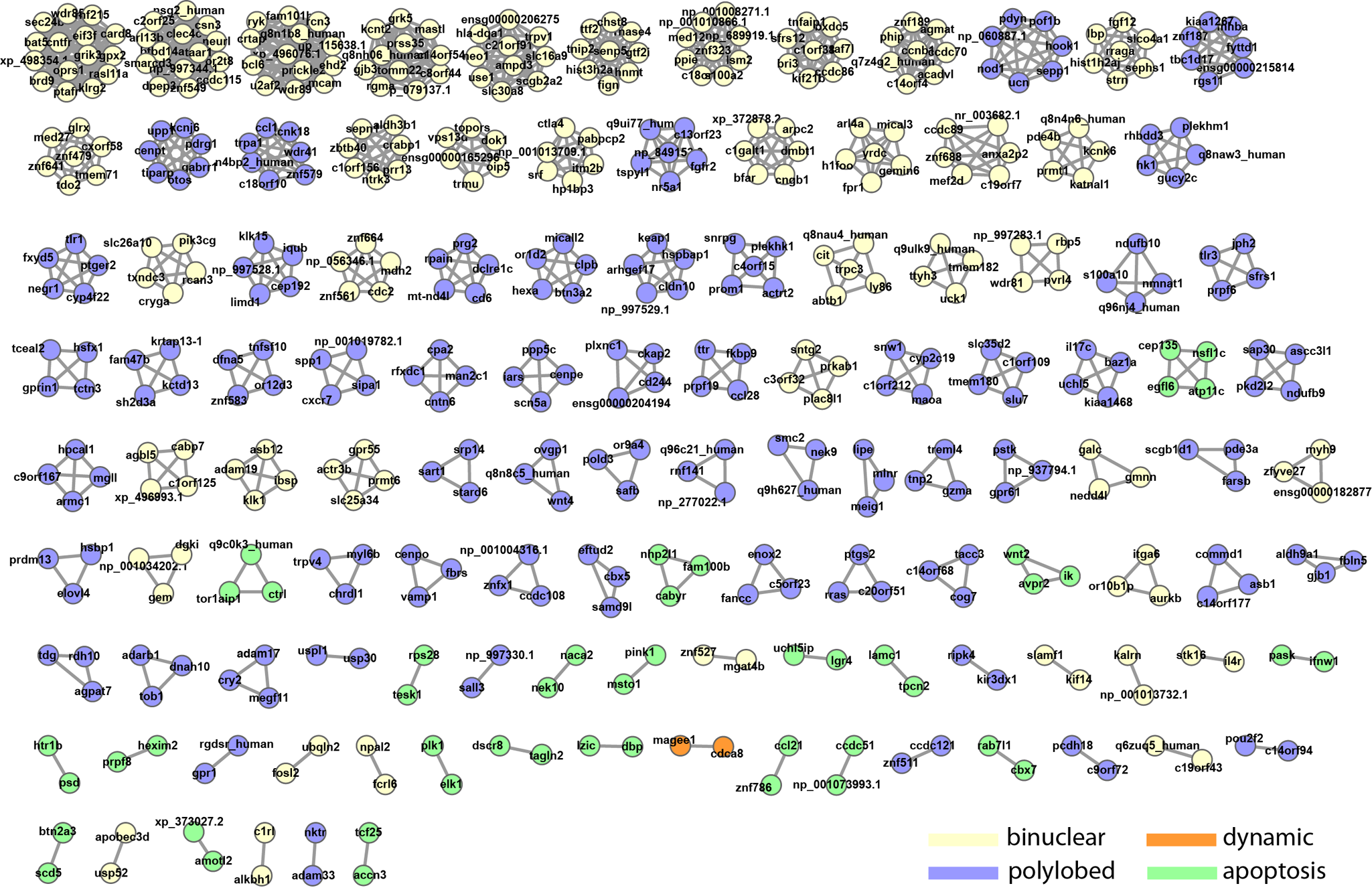

Supplement: Figure S6 — The hypothesized network of synchronously activated genes or proteins involved in the same pathway. The nodes correspond to silenced genes and the genes are connected if they show the exact same phenotypic succession events upon knockdown. The genes are colored according to the first phenotype shown in the cells upon knockdown. Out of all interactions hypothesized, 62.4% have been validated from the literature using GeneMania, with the following distribution: co-expression 64.24%, physical interactions 14.68%, genetic interactions 11.16%, co-localization 5.46%, predicted 4.37%, shared protein domains 0.09%. The networks were visualized using Cytoscape. (TIF) [file pone.0072361.s006.tif]

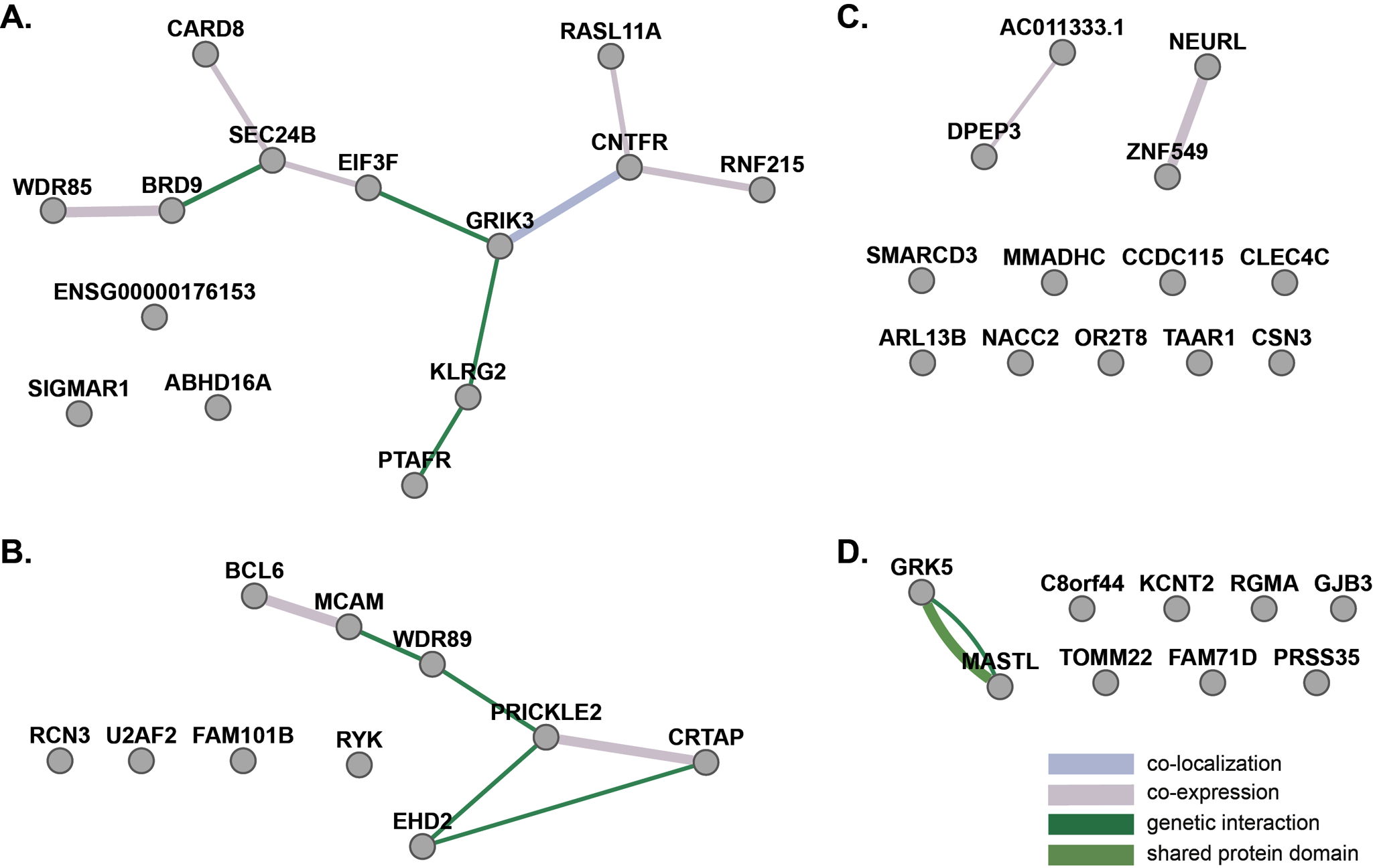

Supplement: Figure S7 — Connections from the literature between genes of four hypothesized interactive modules. The cells where these genes are knocked down adopt a binuclear phenotype after: (A) 16.5 hours; (B) 15 hours; (C) 15.5 hours; (D) 26 hours. The networks were retrieved from GeneMania. (TIF) [file pone.0072361.s007.tif]

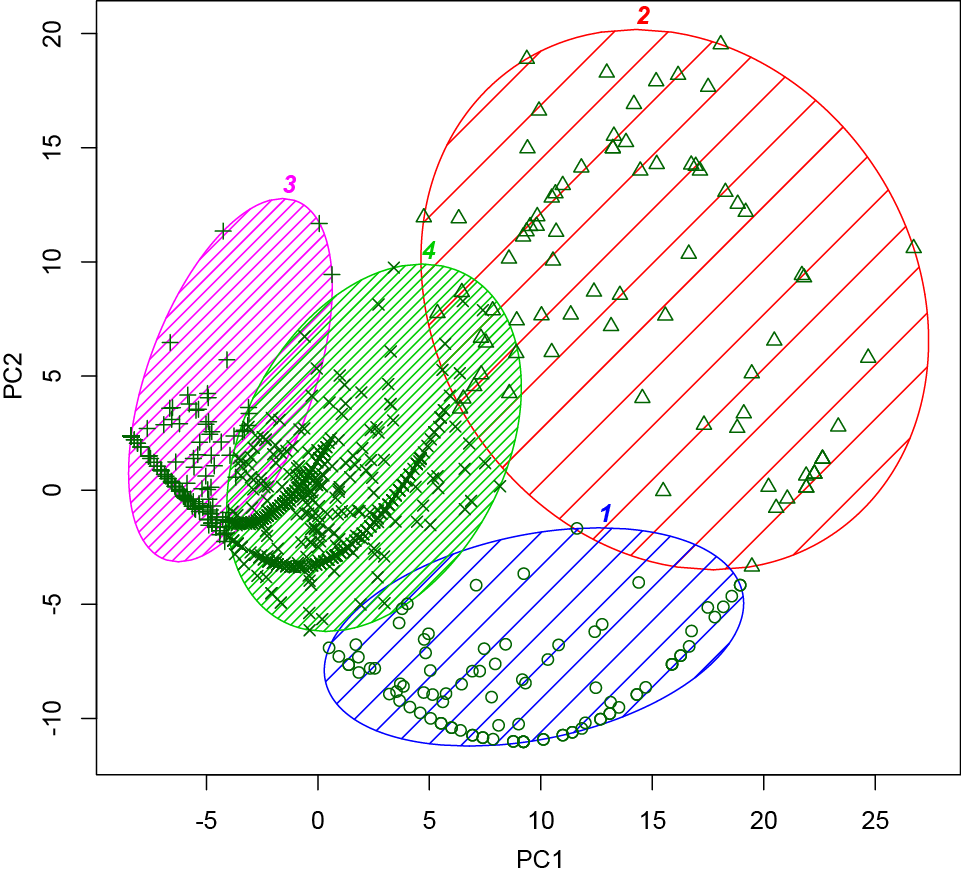

Supplement: Figure S8 — K-means clustering reveals 4 clusters of genes with similar phenotypic succession profiles. The clustering for the first two principal components is displayed. The clustering was performed on the vectors of phenotypic assignment of most prevalent phenotype at each time point for every gene. The clustering and plotting were performed in R. (TIF) [file pone.0072361.s008.tif]

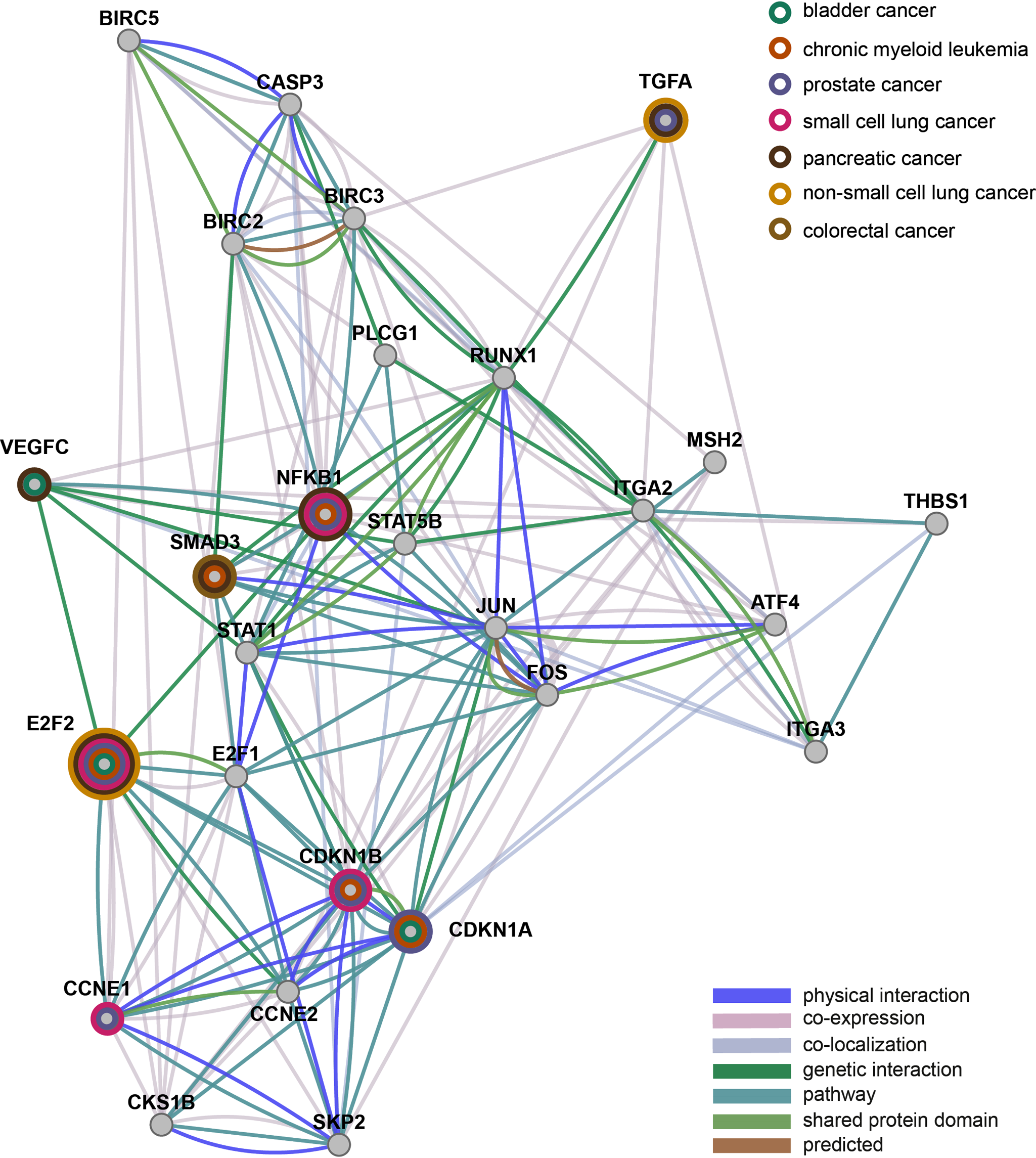

Supplement: Figure S9 — The network of genes affected in cancer that are also periodically transcribed throughout the cell cycle. The genes that related to more than one type of cancer are highlighted. Circle colors indicate the different cancer types where genes are enriched. Genes highlighted in this way are the ones involved in the connections visualized using PhenoTimer. In general, genes affected in the same cancer types interact physically or genetically. The network was retrieved from GeneMania and further edited in Cytoscape. (TIF) [file pone.0072361.s009.tif]

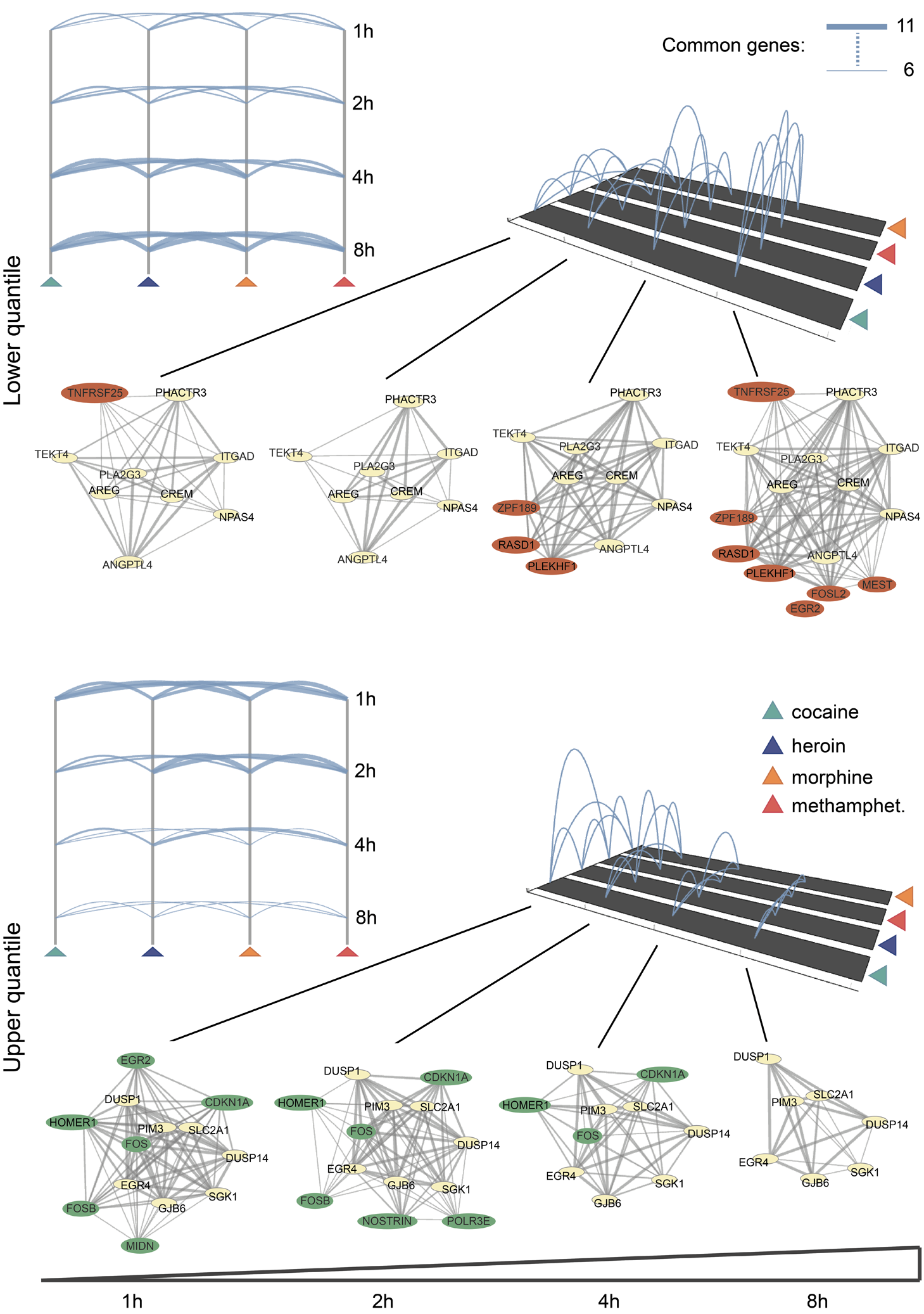

Supplement: Figure S10 — Mechanistic similarities of stimulants (cocaine, methamphetamine) and depressants (heroin, morphine). Action upon genes with transcription profiles in the lower quartile (top) and upper quartile (bottom) ranges is depicted. Two drugs are connected if treatment with either of them results in similar levels of gene expression for at least one gene. The thickness (2D) or height (3D) of the arcs corresponds to the number of genes commonly affected by two drugs. The networks connect genes that respond to the same drug(s). The thickness of the edges corresponds to the number of drugs to which the pair of genes is responsive. Orange nodes (top) and green nodes (bottom) are variable elements, while yellow nodes correspond to the core gene network that stays the same throughout the time course. The plots have been obtained using PhenoTimer and then combined and annotated to emphasize different aspects of the analysis. (TIF) [file pone.0072361.s010.tif]

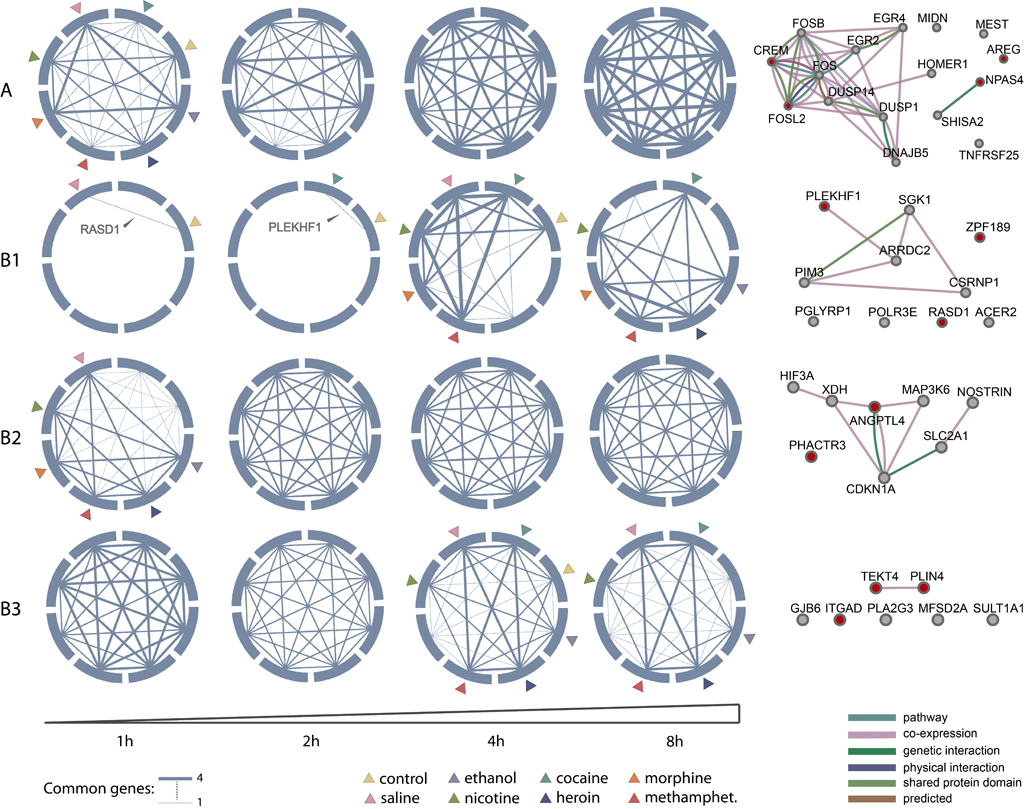

Supplement: Figure S11 — Drug similarities in action on different gene subclasses. Two drugs are connected if they act similarly on at least one common gene, the thickness of the links indicating how many genes are influenced by that pair of drugs. To the right, the networks of the genes corresponding to the different subclasses have been retrieved from GeneMania. The genes highlighted in red appear in drug pair connections. The circular plots have been obtained individually using PhenoTimer and then combined and annotated to emphasize different aspects of the analysis. (TIF) [file pone.0072361.s011.tif]
