# Supplementary material for: PhenoTimer: Software for the Visual Mapping of Time-Resolved Phenotypic Landscapes
Source: PLoS One. 2013 Aug 12;8(8):e72361. doi: 10.1371/journal.pone.0072361 (PMC3741141; doi:10.1371/journal.pone.0072361)
Supplement: Table S1 — Comparison of the different view modes of PhenoTimer. The table lists the comparative strengths and weaknesses of the different graphical representations used in PhenoTimer. (DOC) [file pone.0072361.s012.doc]

| **Graphical representation** | **Strengths** | **Weaknesses** |
| --- | --- | --- |
| 3D arcs | - interactive: zooming, panning, rotating and arc selection capabilities - height is a better indicator of number of genes involved in an arc | - distortion of perspective - occlusion of arcs |
| 2D arcs | - no distortion of perspective - time course patterns can be easily observed | - possible occlusion of arcs by superimposition - the width of the arcs may not be easily comparable |
| circular | - inspection of phenotypic connections for a single time point - no distortion of perspective | - overlapping arcs are not easily spotted - the patterns over the entire time course are more difficult to observe |
| heat map | - overview of all time course profiles, for every gene - dynamic clustering of genes according to their profiles at every time point | - tracing connections between phenotypes over the whole time course is not possible |
| line plot | - comparative inspection of trends in expression profiles over all genes | - profiles are not clustered - it is harder to quantify similarities between profiles |
