# Supplementary material for: PhenoTimer: Software for the Visual Mapping of Time-Resolved Phenotypic Landscapes
Source: PLoS One. 2013 Aug 12;8(8):e72361. doi: 10.1371/journal.pone.0072361 (PMC3741141; doi:10.1371/journal.pone.0072361)
Supplement: Table S2 — Example of an input file loadable into PhenoTimer. The first column specifies the gene names, the second column the phenotypes and the subsequent columns list the gene-associated values at each time point. The fields must be separated by white space. (DOC) [file pone.0072361.s013.doc]

| **Gene name** | **Phenotype name** | **Time points** | | | | |
| --- | --- | --- | --- | --- | --- | --- |
| gene1 | phenotype1 | 0.13 | 0.26 | 0.78 | 0.79 | 1.02 |
| gene1 | phenotype2 | 1.56 | 1.34 | 1.48 | 1.67 | 1.93 |
| gene1 | phenotype3 | 0.59 | 0.04 | -0.02 | -0.02 | -0.13 |
| gene1 | phenotype4 | 0.98 | 1.23 | 1.26 | 1.47 | 1.31 |
| gene2 | phenotype1 | -0.84 | -0.25 | -0.11 | -0.01 | 0.12 |
| gene2 | phenotype2 | 0.74 | 1.35 | 0.99 | 0.92 | 0.91 |
| gene2 | phenotype3 | 0.22 | 0.45 | 0.26 | 0.44 | 0.33 |
| gene2 | phenotype4 | -0.89 | -0.19 | 0.63 | 0.84 | 0.92 |
| gene3 | phenotype1 | 0.22 | 0.21 | 0.19 | 0.18 | 0.22 |
| gene3 | phenotype2 | 0.45 | 0.57 | 0.16 | 0.94 | 0.75 |
| gene3 | phenotype3 | 1.22 | 1.15 | 1.17 | 1.25 | 1.35 |
| gene3 | phenotype4 | 3.20 | 2.18 | 1.29 | 1.87 | 2.08 |
