# Supplementary material for: PhenoTimer: Software for the Visual Mapping of Time-Resolved Phenotypic Landscapes
Source: PLoS One. 2013 Aug 12;8(8):e72361. doi: 10.1371/journal.pone.0072361 (PMC3741141; doi:10.1371/journal.pone.0072361)
Supplement: Table S3 — Example of network files loadable into PhenoTimer. (a) Input file containing the GO enrichment specifications: the columns must specify the GO identifiers, the corresponding descriptions, the p-values of the enrichment and the genes that are enriched for each category, separated by “|”. (b) Along with the enrichment file, an interaction file should also be loaded into PhenoTimer, specifying the interaction partners in the network, one pair per line. The format is the same for other types of networks (e.g. PPIs, metabolic etc.). All these are tab-separated fields. (DOC) [file pone.0072361.s014.doc]

| ***a. GO Enrichment*** | | | | ***b. Interactions*** | |
| --- | --- | --- | --- | --- | --- |
| **GO ID** | **GO name** | **p-value** | **Genes enriched** | **Partner 1** | **Partner 2** |
| 32653 | mitosis | 0.0015 | gene1|gene2|gene4 | 32653 | 54588 |
| 54588 | nuclear division | 0.0023 | gene2|gene5 | 13857 | 32653 |
| 13857 | metaphase plate congression | 0.0001 | gene1|gene3 | 13857 | 54588 |
