# Supplementary material for: PhenoTimer: Software for the Visual Mapping of Time-Resolved Phenotypic Landscapes
Source: PLoS One. 2013 Aug 12;8(8):e72361. doi: 10.1371/journal.pone.0072361 (PMC3741141; doi:10.1371/journal.pone.0072361)
Supplement: Table S4 — GO enrichment for the network of hypothesized synchronous genes. The table lists the molecular functions of all the genes in the mitotic progression dataset whose knockdown causes identical phenotypic successions to at least one other gene. (DOC) [file pone.0072361.s015.doc]

| **GO annotation** | **Q-value** | **Genes in networks** | **Genes in genome** |
| --- | --- | --- | --- |
| catalytic step 2 spliceosome | 1.820605E-3 | 12 | 80 |
| spliceosomal complex | 4.184084E-3 | 13 | 109 |
| nuclear mRNA splicing, via spliceosome | 7.688298E-2 | 15 | 196 |
| RNA splicing, via transesterification reactions with bulged adenosine as nucleophile | 7.688298E-2 | 15 | 196 |
| RNA splicing, via transesterification reactions | 8.786896E-2 | 15 | 202 |
| mRNA processing | 8.967626E-2 | 17 | 256 |
| spliceosome assembly | 1.275452E-1 | 6 | 32 |
| nuclear body | 1.291282E-1 | 11 | 123 |
| ribonucleoprotein complex assembly | 1.491444E-1 | 9 | 85 |
| ribonucleoprotein complex subunit organization | 1.927164E-1 | 9 | 89 |
