# Supplementary material for: PhenoTimer: Software for the Visual Mapping of Time-Resolved Phenotypic Landscapes
Source: PLoS One. 2013 Aug 12;8(8):e72361. doi: 10.1371/journal.pone.0072361 (PMC3741141; doi:10.1371/journal.pone.0072361)
Supplement: Table S5 — Quartile calculations for the measured transcriptional levels upon drug intake. The table lists the quartiles of the normalized and log2-transformed mRNA abundance measured for each drug treatment. The lower (25%) and upper (75%) quartile values are used as thresholds for subsequent visualization and analysis. (DOC) [file pone.0072361.s016.doc]

| **Drug treatment** | **Quartiles** | | | | |
| --- | --- | --- | --- | --- | --- |
|  | **0%** | **25%** | **50%** | **75%** | **100%** |
| Control | 6.376778 | 7.182875 | 7.746917 | 8.936444 | 12.368333 |
| ­Saline | 6.353444 | 7.358917 | 7.830667 | 9.148375 | 12.523333 |
| Ethanol | 6.487556 | 7.410542 | 8.135417 | 9.358958 | 12.667000 |
| Nicotine | 6.449667 | 7.326972 | 7.905417 | 9.147083 | 12.490333 |
| Cocaine | 6.286667 | 7.300000 | 7.972083 | 9.347958 | 12.610667 |
| Heroin | 6.392889 | 7.413083 | 8.165917 | 9.371813 | 12.531667 |
| Morphine | 6.504444 | 7.386278 | 8.114833 | 9.446313 | 12.745667 |
| Methamphetamine | 6.335889 | 7.466917 | 8.108417 | 9.417646 | 12.595000 |
