# Supplementary material for: PhenoTimer: Software for the Visual Mapping of Time-Resolved Phenotypic Landscapes
Source: PLoS One. 2013 Aug 12;8(8):e72361. doi: 10.1371/journal.pone.0072361 (PMC3741141; doi:10.1371/journal.pone.0072361)
Supplement: Table S6 — The functionality description of core network and variable genes similarly regulated by drugs. Lowly and highly expressed genes are defined as before. The descriptions were taken from UniProt. (DOC) [file pone.0072361.s017.doc]

|  | | **Gene** | **Description** |
| --- | --- | --- | --- |
| **Core network** | ***Lowly expressed*** | *Angptl4* | induced under hypoxic conditions in endothelial cells; target of peroxisome proliferation activators |
|  |  | *Areg* | autocrine growth factor as well as a mitogen for a broad range of target cells |
|  |  | *Crem* | transcriptional regulator that binds the cAMP response element |
|  |  | *Itgad* | receptor for ICAM3 and VCAM1; role in atherosclerosis |
|  |  | *Npas4* | transcriptional activator in the presence of ARNT |
|  |  | *Phactr3* | nuclear scaffolding in proliferating cells |
|  |  | *Pla2g3* | catalyzes the calcium-dependent hydrolysis of the 2-acyl groups in 3-sn-phosphoglycerides |
|  |  | *Plekhf1* | may induce apoptosis through the lysosomal-mitochondrial pathway |
|  |  | *Rasd1* | small GTPase |
|  |  | *Tekt4* | structural component of ciliary and flagellar microtubules |
|  | ***Highly expressed*** | *Dusp1* | dual specificity phosphatase that dephosphorylates MAP kinase MAPK1/ERK2 |
|  |  | *Dusp14* | involved in the inactivation of MAP kinases |
|  |  | *Egr4* | transcriptional regulator |
|  |  | *Gjb6* | gap junction protein |
|  |  | *Homer1* | postsynaptic density scaffolding protein |
|  |  | *Pim3* | proto-oncogene with serine/threonine kinase activity |
|  |  | *Sgk1* | serine/threonine-protein kinase with important role in cellular stress response |
|  |  | *Slc2a1* | facilitative glucose transporter |
| **Variable genes** | ***Lowly expressed*** | *Egr2* | sequence-specific DNA-binding transcription factor |
|  |  | *Fosl2* | controls osteoclast survival and size |
|  |  | *Hif3a* | involved in adaptive response to hypoxia |
|  |  | *Mest* | mesoderm specific transcript |
|  |  | *Tnfrsf25* | mediates activation of NF-kappa-B and induces apoptosis |
|  |  | *Zfp189* | may be involved in transcriptional regulation |
|  | ***Highly expressed*** | *Cdkn1a* | binds to and inhibits cyclin-dependent kinase activity, blocking cell cycle progression |
|  |  | *Fos* | role in signal transduction, cell proliferation and differentiation |
|  |  | *Fosb* | interacts with Jun proteins enhancing their DNA binding activity |
|  |  | *Egr2* | sequence-specific DNA-binding transcription factor |
|  |  | *Midn* | may be involved in regulation of genes related to neurogenesis in the nucleolus |
|  |  | *Nostrin* | endothelial nitric oxide synthase traffic inducer |
|  |  | *Polr3e* | DNA-directed RNA polymerase III 80 kDa polypeptide |
|  |  | *Sult1a1* | thermostable phenol sulfotransferase |
